# HER2 amplification level is not a prognostic factor for HER2-positive breast cancer with trastuzumab-based adjuvant treatment: a systematic review and meta-analysis

**DOI:** 10.18632/oncotarget.11541

**Published:** 2016-08-23

**Authors:** Qian-Qian Xu, Bo Pan, Chang-Jun Wang, Yi-Dong Zhou, Feng Mao, Yan Lin, Jing-Hong Guan, Song-Jie Shen, Xiao-Hui Zhang, Ya-Li Xu, Ying Zhong, Xue-Jing Wang, Yan-Na Zhang, Qiang Sun

**Affiliations:** ^1^ Department of Breast Surgery, Peking Union Medical College Hospital, Chinese Academy of Medical Sciences and Peking Union Medical College, Beijing 100730, P. R. China

**Keywords:** HER2-positive breast cancer, HER2 amplification, prognosis, trastuzumab-based treatment, adjuvant setting

## Abstract

**Background:**

Trastuzumab-based therapy is a standard, targeted treatment for HER2-positive breast cancer in the adjuvant setting. However, patients do not benefit equally from it and the association between HER2 amplification level and patients' survival remains controversial. A systematic review and meta-analysis was conducted by incorporating all available evidence to evaluate the association between disease free survival (DFS) and HER2 amplification level.

**Results:**

Three cohort studies involving 1360 HER2-positive breast cancer patients stratified by HER2 amplification magnitude were eligible for meta-analysis. The combined HRs for DFS were 1.05 (95% CI: 0.80−1.36, *p* = 0.74) and 0.97 (95% CI: 0.73−1.29, *p* = 0.83) for HER2 gene copy number (GCN) and HER2/CEP 17 ratio. No evidence of heterogeneity or public bias was found.

**Methods:**

Databases including PubMed, Embase, Web of Science, and Cochrane Central Register of Controlled Trials (CENTRAL), were searched for eligible literature. HER2 amplification level was evaluated by fluorescence *in situ* hybridization (FISH) in terms of gene copy number (GCN) and HER2/CEP17 ratio. Hazard ratios (HRs) for DFS with 95% confidence interval (CI) according to the amplification level of HER2 were extracted. The outcomes were synthesized based on a fixed-effects model.

**Conclusions:**

HER2 amplification level is not a prognostic factor for HER2-positive breast cancer with trastuzumab-based targeted therapy in the clinical adjuvant setting.

## INTRODUCTION

The human epidermal growth factor receptor 2 (HER2) is a 185-kd glycoprotein with tyrosine kinase activity. HER2 protein overexpression or gene amplification (HER2 positivity) occurs in approximately 20% to 30% of newly diagnosed invasive breast cancers. HER2 positivity has been shown to confer an adverse prognosis [1–3] and serve as a predictor of clinical response to treatment with the humanized monoclonal antibody trastuzumab [4, 5]. Trastuzumab shows considerable clinical efficacy and extends the overall survival of certain patients with HER2-positive breast cancer [6–10]. However, heterogeneity exists within HER2-positive tumors, and the overall response rate to trastuzumab-containing therapies remains modest-approximately 26% when used as a single therapy and 40% to 60% when used in combination with systemic chemotherapy [8, 10, 11].

Studies have been conducted to investigate the relationship between HER2 status and response to trastuzumab or clinical outcomes in HER2-positive breast cancer cohorts with trastuzumab-containing treatment. All related studies are summarized in Table 1 [12–39]. All included participants were HER2-positive breast cancer patients who received trastuzumab-based therapy according to the standard dose regimen. HER2 status of primary tumor or serum was assessed at a minimum of one of three levels including DNA, RNA, and protein. In a neoadjuvant setting, both DNA amplification and mRNA expression positively correlated with response to trastuzumab[40–42]. There was a discrepancy at the protein level in the two studies, which used different scoring systems and found that the Hercept test scoring system may be not accurate enough to identify the correlation [40, 41]. In metastatic setting, seven out of eight studies at DNA and mRNA level showed there was a positive correlation between HER2 amplification or mRNA expression level and response to trastuzumab or survival [43–49]. The only study that did not reach the limit of statistical significance was an explorative study with a small sample size of 33 [50]. Quantitative assessments of the HER2 protein level were done in both primary surgical specimen and serum samples. Serial monitoring of HER2 protein in serum after the beginning of trastuzumab treatment revealed that the decreased extent of HER2 concentration value was positively correlated with response and survival [51–54]. Expression level of HER2 protein or activated p-HER2 in tumor tissue was positively correlated with response and survival [43, 46, 47, 55–58]. Tumors that expressed p95HER2, an amino terminally truncated HER2 receptor that lacks the extracellular trastuzumab-binding domain, were resistant to trastuzumab and have a shorter progression free survival (PFS) and overall survival (OS) [59, 60]. In other words, HER2 level seems to be a positive predictive and prognostic factor for HER2-positive breast cancer in neoadjuvant or metastatic setting.

**Table 1:** Correlations between HER2 levels and response to trastuzumab or survival

Neoadjuvant setting						
Level	Sample	Study	Population	Method	Result	Conclusion
**DNA**	tissue	Laurent Arnould/2007 Clin cancer res	HER2 (+) locally advanced BC T-based therapy	FISH	High HER2 amplification level----high pCR rate	Positive correlation with response
		S Guiu/2010 British journal of cancer	HER2 (+) BC T-based therapy	FISH	High HER2 amplification level----high pCR rate The level of HER2 gene amplification did not influence RFS or OS.	Positive correlation with response No correlation with survival
**mRNA**	tissue	Andreas Schneeweiss/2014 Breast cancer research	HER2 (+) EBC T plus pertuzumab	qRT-PCR	High HER2 mRNA expression level----high pCR rate	Positive correlation with response
**Protein**	tissue	Laurent Arnould/2007 Clin cancer res	HER2 (+) locally advanced BC T-based therapy	IHC, HercepTest scoring system (0, 1+, 2+, 3+)	No correlation with pCR rate	No correlation with response
		Andreas Schneeweiss/2014 Breast cancer research	HER2 (+) EBC	IHC, H-Score (0~400)	High HER2 protein expression level----high pCR rate	Positive correlation with response
**Adjuvant setting**						
**DNA**	tissue	S. Paik/2007 Journal of Clinical Oncology	HER2 (+) BC T-based therapy	FISH	No correlation with DFS	No correlation with survival
		Mitch Dowsett/2009 Journal of clinical oncology	HER2 (+) BC T-based therapy	FISH	No correlation with DFS	No correlation with survival
		Edith A. Perez/2010 Journal of clinical oncology	HER2 (+) BC T-based therapy	FISH	No correlation with DFS	No correlation with survival
		A Borley/2014 British journal of cancer	HER2 (IHC2 + FISH +) BC T-based therapy	FISH	High and low level HER2 amplification levels----longer DFS than intermediate level	Parabolic correlation with survival
		Qijia Xuan/2015 Breast cancer res treat	HER2 (+) BC T-based therapy	FISH	High HER2 amplification level----shorter DFS	Negative correlation with survival
**Protein**	tissue	S. Paik/2007 Journal of Clinical Oncology	HER2 (+) BC T-based therapy	IHC, HercepTest scoring system (0, 1+, 2+, 3+)	No correlation with DFS	No correlation with survival
		H. Joensuu/2011 Annals of oncology	HER2 (+) EBC T-based therapy	HERmark H2T	Very high tumor HER2 content (log H2T ≥ 2.1)----less benefit from T	Negative correlation with benefit
**Metastatic setting**						
**DNA**	tissue	Rosa Giuliani/2007 European journal of cancer	HER2 (+) MBC T-based therapy	FISH	Increased HER2/CEP17 ratio----high response rate	Positive correlation with response
		P. A. Kaufman/2007Journal of Clinical Oncology	HER2 (+) MBC T-based therapy	FISH	High amplification level----higher response rate	Positive correlation with response
		Giuseppe Gullo/2009 Invest new drugs	HER2 (+) MBC T-based therapy	FISH	High HER2 amplification level----shorter TTP and OS(no statistical significance)	No correlation with survival
		Hye-Suk Han/2009 J Korean Med Sci	HER2 (FISH+) MBC first-line T-based therapy	FISH	High HER2 amplification level----longer TTP	Positive correlation with survival
		Ji-Won Kim/2013 Cancer chemother pharmacol	HER2 (FISH+) MBC first-line T-based therapy	FISH	Patients with a HER2/CEP17 ratio ≥ 3.0 had significantlylonger PFS with a tendency toward a higher RR and longer OS	Positive correlation with survival and response
		Eva-Maria Fuchs/2014 Int. J. cancer	HER2 (+) MBC T-based therapy	FISH	High level HER2 amplification---longer PFS and improved CR and PR	Positive correlation with survival and response
**RNA**	tissue	Maria vassilakopoulou/2014 Plos one	HER2 (+) MBC T-based therapy	RNAscope, AQUA	High HER2 mRNA level----longer TTP survival and OS	Positive correlation with survival
		Edith A perez/2014 Breast cancer research	HER2 (+) MBC HT or T-DM1	qRT-PCR	High HER2 mRNA----enhanced improved PFS with T-DM1 relative to HT(trastuzumab plus docetaxel)	Positive correlation with survival
**protein**	tissue	Maurizio Scaltriti/2007 J Natl Cancer Inst	HER2 (+) MBC T	Immunofluorescence P95HER2	Breast tumors that express p95HER2 were resistant to T.	Negative correlation with response
		Rosa Giuliani/2007 European journal of cancer	HER2 (+) MBC T-based therapy	IHC p-HER2	Activated p-HER2----high RR	Positive correlation with response
		Christine Desmedt/2009 Diagn mol pathol	HER2 (+) MBC T-based therapy	VeraTag assay Expression and dimer	High HER2 expression or HER2:HER2 dimer levels----longer TTP	Positive correlation with survival
		Jeff Sperinde/2010 Clin cancer res	HER2 (+) MBC T	VeraTag FFPE assay p95HER2	High p95HER2 expressing level----shorter PFS and OS	Negative correlation with survival
		Allan Lipton/2010 cancer	HER2 (+) MBC T-based therapy	HERmark H2T	High HER2 expressing level----longer TTP	Positive correlation with survival
		Masakaz Toil/2010 BMC cancer	HER2 (+) MBC T-based therapy	HERmark H2T	High HER2-expressing group: higher H2T level----longer OS Low HER2-expressing group: lower H2T level----longer OS	Positive correlation with survival in high HER2-expressing group
		M.Bates/2011 Annals of oncology	HER2 (+) MBC T-based therapy	HERmark H2T	Parabolic relationship: TTP improved with increasing H2T until, at the highest levels of H2T, an abrupt decrease in the TTP was observed.	Positive correlation with survival except the highest-level subgroup
		Ji-Won Kim/2013 Cancer chemother pharmacol	HER2 (FISH+) MBC first-line T-based therapy	IHC, HercepTest scoring system (0, 1+, 2+, 3+)	Patients with HER2 IHC 1+ had significantly shorter OS, PFS and lower RR than IHC2+/3+.	Positive correlation with survival
		Maria vassilakopoulou/2014 Plos one	HER2 (+) MBC T-based therapy	Immunofluorescence ICD, ECD	High ICD and ECD expression----longer TTP and OS	Positive correlation with survival
	serum	Wolfgang J. Kostler/2004 Clinical cancer research	HER2 (+) MBC T-based therapy	ELISA	1. High baseline level----high response rate 2. Serial monitoring: 1)Decreased ECD value----increased probability of response, 2) less decrease from baseline HER2 concentration at day 15 ----shorter PFS	Decreased HER2 concentration value----positive correlation with response and survival
		Francisco J Esteva/2005 Breast cancer research	HER2 (+) MBC T-based therapy	Bayer Immuno1 or ADVIA Centaur^™^ automated assay	1. Baseline HER2 concentrations----no correlation with PFS 2. Serial monitoring: less decrease from baseline HER2 concentration in early weeks after start of therapy----shorter PFS.	Decreased HER2 concentration value----positive correlation with survival
		Sun-Young Kong/2006 Cancer res treat	HER2 (+) MBC T-based therapy	ADVIA Centaur immunoassay system	1. Baseline HER2 concentrations---no correlation with response rate 2. Serial monitoring: Decrease ECD value----increased probability of response	Decreased HER2 concentration value----positive correlation with response
		S. M. Ali/2006 Journal of Clinical Oncology	HER2 (FISH+) MBC first-line T-based therapy	ELISA	Patients with < 20% decrease in serum HER-2/neu levels have decreased benefit(ORR, DRP, TTP, OS) from T therapy	Decreased HER2 concentration value----positive correlation with response and survival

Abbreviations: BC: breast cancer, DRP: duration of response, EBC: early breast cancer, ECD: HER2 extracellular domain, H2T: total HER2 content, ICD: HER2 intracellular domain, RFS: recurrence-free survival.

However, results from previous studies in adjuvant setting were controversial; three studies showed no correlation [61–63], one showed parabolic correlation [64], and one showed a negative correlation between HER2 amplification and clinical survival [65]. There was also no accordance for the relationship at the protein level, although different testing methods and scoring systems were used. These outcomes raised the question: Could HER2 amplification level assessed by FISH serve as a prognostic biomarker for clinical HER2 positive patients who receive standardized trastuzumab targeted therapy in an adjuvant setting? In this study, we performed a systematic review and meta-analysis by incorporating all availableevidence to evaluate the DFS according to HER2 amplification level in patients with HER2-positive breast cancer.

## RESULTS

### Study selection

**Figure 1:**
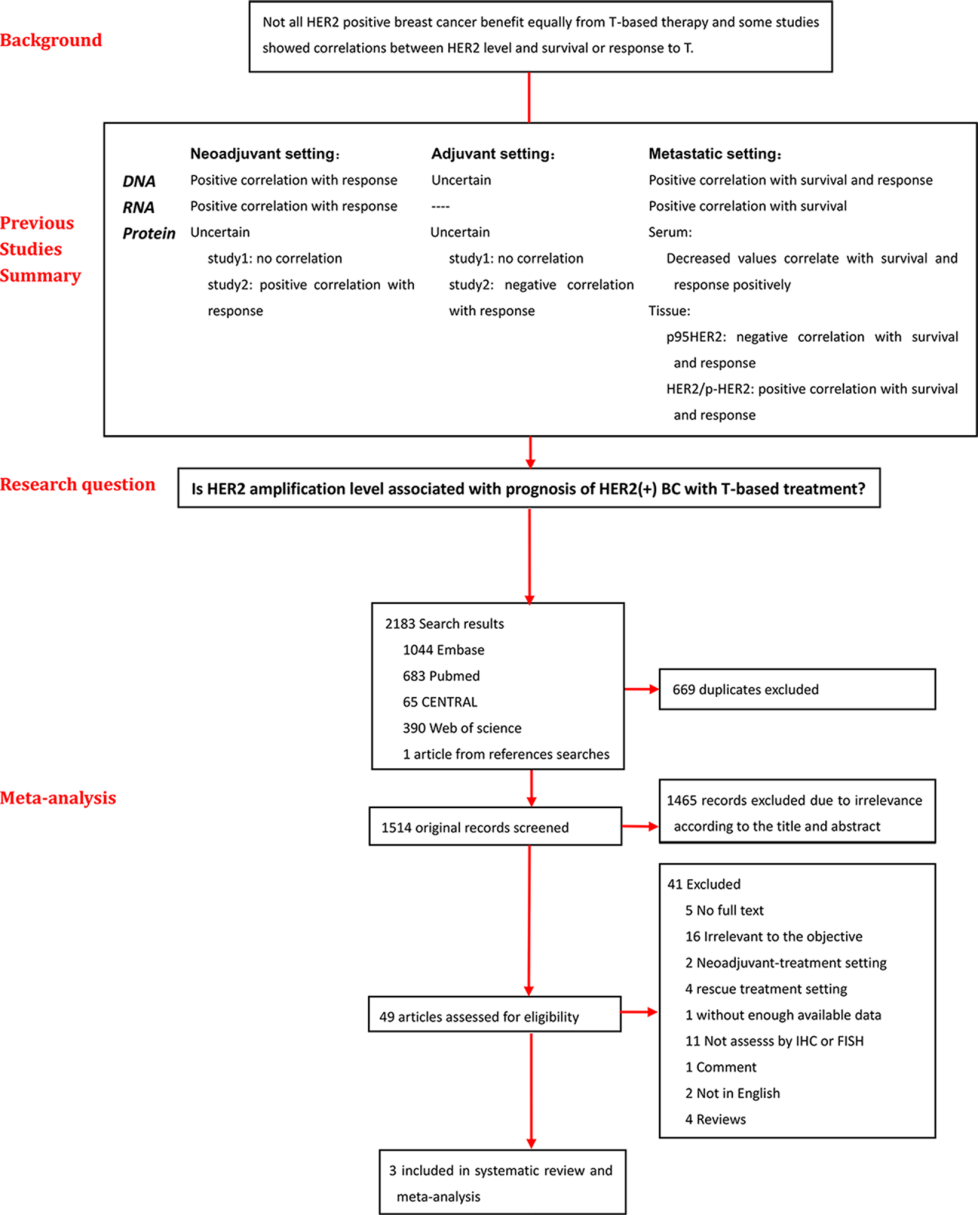
Flow chart of the research Firstly, all available studies related with relationship between HER2 levels including DNA, RNA, and protein and clinical response to trastuzumab-containing therapies or survival were reviewed and summarized. Then a meta-analysis was performed in 3 studies to draw the relationship between HER2 amplification level and prognosis of HER2 positive breast cancer with trastuzumab-base therapy in adjuvant setting. Abbreviations: T-based, trastuzumab-based; BC, breast cancer.

Figure 1 illustrates the flow diagram of candidate selection records in our study. The search retrieved 2,183 articles, of which 669 duplicates were identified. Of 1,514 remaining articles, 1,465 were excluded based on title and abstract reviewing and 49 references remained to achieve full text for further screening. Of these, full text was not available for five, 16 were not relevant, six were in the neoadjuvant or metastatic setting, one did not provide enough data, 11 assessed HER2 status with methods other than IHC or FISH, seven were reviews or comments or in other language, and only three satisfied inclusion criteria.

### Characteristics of included studies

The main characteristics of the included studies are displayed in Table 2 [66–68]. The studies were conducted all over the world, including Europe, America, Asia, and Africa. Two were retrospective cohort studies and the other was a prospective cohort within a large international randomized clinical trial. The trastuzumab-based therapy applied in different studies was single trastuzumab therapy or in combination with traditional chemotherapy (mainly paclitaxel). All the studies adopted the immunohistochemistry (IHC) or FISH method to assess the HER2 status. The number of patients stratified by HER2/CEP17 ratio or (and) HER2 GCN ranged from 59 to 1000. The follow-up period was long enough for the outcomes to occur. All the studies used DFS as the end point. The HER2 amplification was divided into high and low levels according to the original article and corresponding HRs for high to low HER2 amplification level are shown in Table 2. None of these three studies gained a Newcastle-Ottawa Quality Assessment Scale (NOS) < 6, suggesting that all of them have high level of methodological quality in this meta-analysis.

**Table 2:** Characteristics of studies included in the meta-analysis and the extracted HRs

Study	Country	Study type	Median age	Follow-up period	HER2 positive criterion	Stratus of HER2 level	Number of patients stratified by HER2 amplification	Treatment regiment	Endpoint	HRs (95% CI)	Study quality
Mitch Dowsett/2009 J Clin Oncol	Argentina, Australia, Austria, et ala.	Prospective cohort within Randomized trial (HERA)	49	Median: 2 years (0, 48months)	IHC3+ or/and FISH amplified IHC: at least 10% invasive cells with complete membrane staining FISH: HER2/CEP17 ratio ≥ 2.0	GCN: Low: 4 ≦ HER2 GCN ≦ 13 High: 13 < HER2 GCN FISH ratio: Low: 2≦ FISH ratio ≦6 High: 6 < FISH ratio	1000	Control arm: chemotherapy →observation Test arm: chemotherapy →1 year T	DFS	GCN: 1.03 (0.86, 1.23) Ratio: 1.00 (0.83, 1.20)	NOS 7
A Borley/2014 British Journal of Cancer	UK	Retrospective cohort study	59	7years	IHC2+ and FISH amplified, FISH: HER2/CEP17 ratio > 2.0	GCN: Low: 2 ≦ HER2 GCN ≦ 12 High: 12 < HER2 GCN	59	T-based therapy	PFS	3.90 (0.43, 35.21)	NOS 6
Qijia Xuan/2015 Breast Cancer Res Treat	China	Retrospective cohort study	48 (19–74)	60+ months	FISH amplified: a HER2/CEP17 ratio ≥ 2.0 or an HER2/CEP17 ratio < 2.0 with an average HER2 GCN > 6.0 signals/cell	GCN: GCN < 11.5 GCN ≥ 11.5 FISH ratio: FISH ratio < 6.5; FISH ratio ≥ 6.5	291	T-based therapy	DFS	GCN: 1.06 (0.53, 2.12) Ratio: 1.15 (0.59, 2.25)	NOS 8

a Belgium, Brazil, Canada, Chile, China, Colombia, Croatia, Denmark, France, Germany, Greece, Guatemala, Hong Kong, Hungary, Ireland, Israel, Italy, Japan, Korea, Republic of, Netherlands, Poland, Portugal, Russian Federation, Singapore, South Africa, Spain, Sweden, Switzerland, Thailand, United Kingdom.

### HER2 GCN and survival

**Figure 2:**
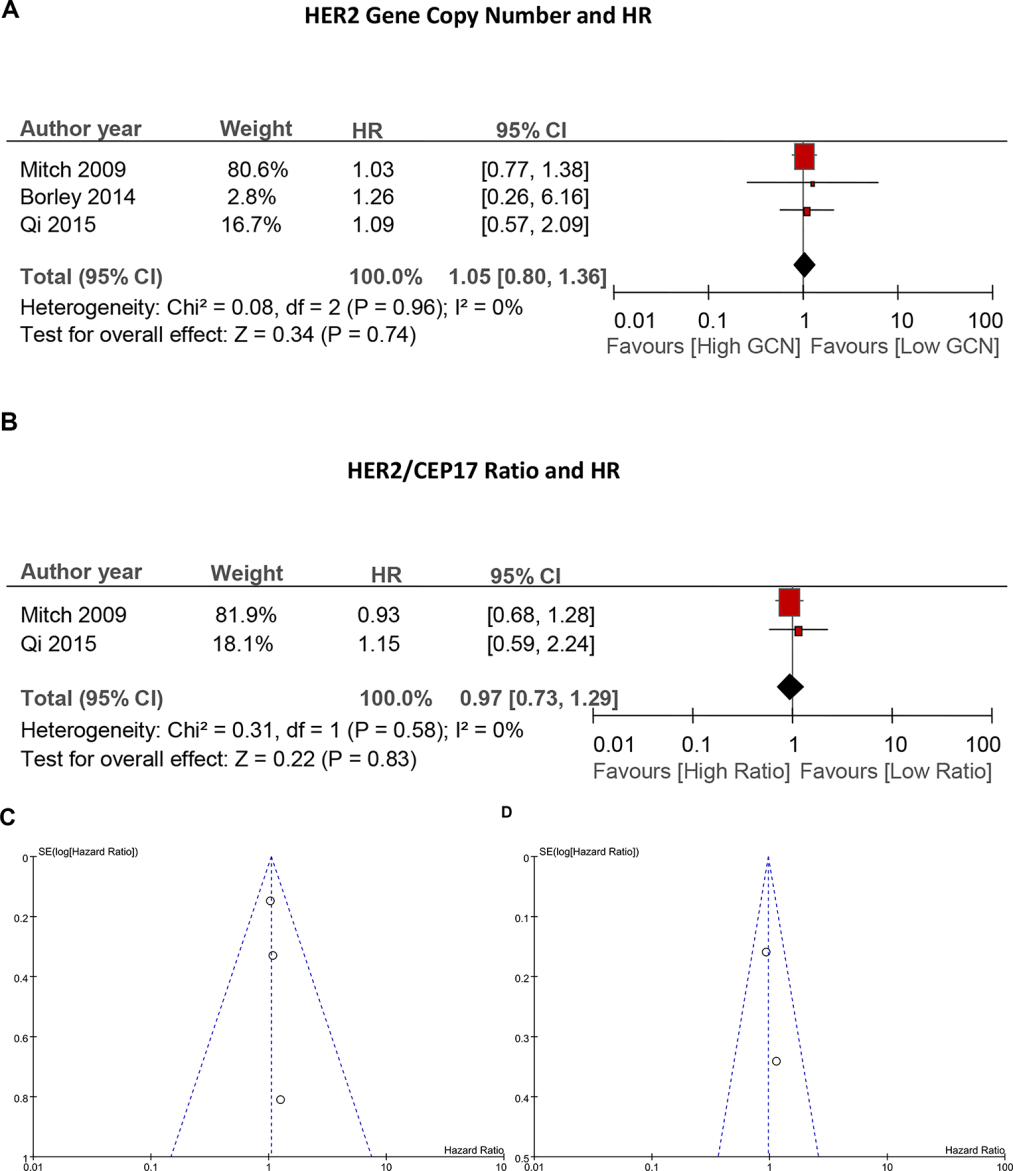
Forest plots and funnel plots Forest plots showing relationship between HER2 amplification level and survival: (**A**) showing no relationship between HER2 gene copy number with DFS, and (**B**) showing no relationship between HER2/CEP17 ratio with DFS. Funnel plots for the evaluation of potential publication bias in the impact of HER2 GCN (**C**) or HER2/CEP17 ratio (**D**) on DFS with HER2 positive breast cancer.

As shown in Figure 2A, there was no heterogeneity among these studies (I^2^ = 0%, *P* = 0.96). The fixed-effects model analysis showed that, the overall HR was 1.05 (95% CI: 0.80–1.36, *p* = 0.74), indicating that different HER2 GCN level had no prognostic value on the trastuzumab-based treatment outcomes in HER2-positive breast cancer patients.

### HER2/CEP17 ratio and survival

Two studies assessed the clinical benefit from trastuzumab-based adjuvant therapy upon different HER2/CEP17 ratio. Without heterogeneity (I^2^ = 0%, *P* = 0.58), a combined HR and its 95% CI were calculated with a fixed-effects model. The results did not show a statistically significant relationship between HER2/CEP17 ratio and survival (HR = 0.97, 95% CI: 0.73–1.29, *p* = 0.83) (Figure 2B) either.

### Publication bias

No evidence of publication bias was indicated (Figure 2C and Figure 2D) in this study. However, because of the limited number of included studies, whether publication bias exists in the current meta-analysis is still difficult to confirm.

## DISCUSSION

This is a systematic review and meta-analysis to evaluate the prognostic value of HER2 gene amplification level in HER2-positive breast cancer patients undergoing trastuzumab-based adjuvant therapy after radical surgery treatment. Three studies assessed HER2 GCN and two studies assessed HER2/CEP17 ratio. Results showed that neither HER2 GCN nor HER2/CEP17 ratio had an impact on prognosis in the adjuvant treatment stage for HER2-positive breast cancer. In other words, different HER2 amplification level does not associate with HER2-positive breast cancer prognosis in the adjuvant setting.

HER2 status is closely correlated with a therapeutic response to anti-HER2 treatment in neoadjuvant and metastatic settings. Previous studies showed higher HER2 amplification level was a favorable predictor for pathologic complete response (pCR) in a neoadjuvant setting [69–71]. In the metastatic setting, increasing values of HER2 amplification were associated with a significantly longer PFS or a significantly higher objective response rate (ORR) [72]. Therefore, it seemed consistent to regard a high level of HER2 amplification as a good predictive biomarker both for early HER2-positive breast cancer in the neoadjuvant treatment stage and HER2-positive MBC and as a prognostic biomarker for MBC. There were also other methodologies, including a quantitative measurement of HER2 protein by Vera Tag assay and assessment of HER2 mRNA by qPCR or RNAscope, that have been applied and evaluated for the predictive value [15–17, 21, 25, 28]. Those studies showed that higher HER2 protein and mRNA expression were significantly associated with higher pCR rate, higher response rate (RR), longer time to progression (TTP), longer PFS and OS in some trastuzumab-treated metastatic cohorts.

However, the relationship between the level of HER2 amplification and efficacy of trastuzumab-based therapy in adjuvant treatment remains controversial. We performed this systematic review and meta-analysis to summarize the results of all the relevant studies. This is the first meta-analysis to summarize the prognostic value of HER2 amplification level for HER2 positive early breast cancer in an adjuvant treatment setting. Results showed that HER2 GCN and HER2/CEP17 ratio did not influence prognosis in patients treated with trastuzumab-containing adjuvant comprehensive treatment. The pCR rate in neoadjuvant cannot be translated to survival benefit. When a tumor metastasizes to distant organs, the biological behavior of metastasis differs from the primary lesion. Therefore, the prognostic value of HER2 amplification level in palliative treatment cannot be applied in adjuvant treatment directly.

Although HER2 positivity indicates worse prognosis for breast cancer [1–3], the results of this meta-analysis indicated that further discriminating differences in HER2 amplification magnitude have insignificant prognostic value in adjuvant setting. It can be inferred that there might be a threshold effect that any degree of HER2 amplification above the cutoff value has similar clinical benefit from trastuzumab treatment. Surgeons and oncologists do not need to pay extra attention to patients' HER2 GCN and HER2/CEP17 ratio when already judged as amplification positive. Consequently, it highlights the importance to assess the HER2 positivity accurately to ensure the potential benefit.

However, significant heterogeneity exists among HER2-positive breast cancer patients as evidenced by the fact that some HER2-positive breast cancer patients exhibit de novo trastuzumab resistance, it is therefore necessary to identify the predictive markers. While mRNA and protein are downstream products of DNA and functionally related, it is reasonable to speculate that quantitative assessment of HER2 mRNA or protein may potentially identify the populations with different response and outcomes. So more studies especially prospective clinical trials concentrated on mRNA or protein level should be conducted in HER2-positive breast cancer population to identify these predictive markers.

This study was attempting to answer the question whether the amplification level of HER2 impacts patients' prognosis with HER2-positive breast cancer. Certain limitations existed and some results needed to be cautiously interpreted. First, we estimated the HR for DFS from Kaplan-Meier curves, which may have compromised the precision of the data. Second, the number of included studies and total sample size are relatively small, which might have impacted the validity of our analysis. The further elaboration is that the included three studies are the total data which are available to analyze and there do exist controversies between the conclusions retrieved from them. The other three studies listed in Table 1 were excluded because of lacking of full-text or analyzable data or being aimed at protein level. No heterogeneity and no evidence of publication bias was found. Third, cut-off values distinguishing high and low levels of HER2 amplification determined by FISH evaluation vary in studies and may have led to between-study heterogeneity.

## MATERIALS AND METHODS

### Search strategy

This meta-analysis was reported according to the Preferred Reporting Items for Systematic Reviews and Meta-analysis (PRISMA) statement [73]. Two authors independently carried out the systematical search for relevant randomized clinical trials and cohort studies in Pubmed, Embase, Web of science and the CENTRAL published before Jun. 19, 2015. Searches were limited to human studies in English, using combinations of the following search terms: “breast neoplasma”, “breast cancer”, “breast carcinoma”, “mammary neoplasma”, “mammary cancer”, “mammary carcinoma”, “genes, erbB-2”, “HER2”, “*In Situ* Hybridization, Fluorescence”, “prognosis” and “treatment outcome”. We also manually reviewed relevant references and reviews.

### Inclusion and exclusion criteria

Studies meeting the following criteria were eligible for the meta-analysis: (1) clinical trials or cohorts with full text; (2) participants within the study were ensured HER2-positive breast cancer patients assessed by IHC or (and) FISH and received trastuzumab no less than 1 year of adjuvant trastuzumab treatment; (3) the level of HER2 amplification was divided into at least two stratums; (4) survival data stratified by HER2 amplification level was available. Studies that failed to fulfill the above inclusion criteria were excluded from this study.

### Outcome definition and data extraction

The primary end point for this meta-analysis was DFS. DFS data was extracted in the form of hazard ratio (HR) with the corresponding 95% confidence interval (CI). All eligible publications were reviewed, and data extraction was done by two independent authors. The following information was extracted: (a) general information, including name of first author, year of publication, country (area) of origin, study type, age of the participants and follow-up period; (b) HER2 positive criterion, method to stratify HER2 amplification level and number of patients stratified by HER2 amplification; (c) treatment regiment and HRs for DFS with 95% confidence intervals.

### Quality assessment

Two independent reviewers assessed the quality of each study with the NOS. A study with NOS > 5 was regarded as a high-quality study. Disparity was resolved by discussion or consultation.

### Statistical analysis

In this meta-analysis, HR and 95% CI were used to assess the association of HER2 amplification level and DFS in patients with HER2 positive breast cancer. We estimated the HR and its 95% CI with a previously validated method described by a previously published article [74]. When an article only had K-M curves, we used Engauge Digitizer, a digitizing program, that could translate curves into numbers to extract survival data from its curves, and then put the data into a spreadsheet, called Tierney table, by which the estimated HR and corresponding 95% CI were calculated immediately. We assessed heterogeneity across studies with the Cochran *Q* test and the *I^2^* statistic. An *I^2^* value less than 50% was regarded as evidence of low heterogeneity, for which the fixed-effects model was utilized to pool the results;. Potential publication bias was assessed by visual inspection of the funnel plots, in which the standard error of logHR of each study was plotted against its logHR. We did not conduct further statistical tests for funnel plot asymmetry because of the limited test power when fewer than 10 studies were included [75]. A *p* value < 0.05 considered significant for all tests. All analyses were conducted using the Review Manager (RevMan) (Version 5.3. Copenhagen: The Nordic Cochrane Centre, the Cochrane Collaboration, 2014).

## CONCLUSIONS

The present meta-analysis indicates that different HER2 amplification level is not associated with trastuzumab-based therapy response and clinical survival. The HER2 amplification should be assessed accurately to ensure enough appropriate treatment for HER2-positive breast cancer.
